# Impact of the Resident Microbiota on the Nutritional Phenotype of *Drosophila melanogaster*

**DOI:** 10.1371/journal.pone.0036765

**Published:** 2012-05-07

**Authors:** Emma V. Ridley, Adam C-N. Wong, Stephanie Westmiller, Angela E. Douglas

**Affiliations:** 1 Department of Biology, University of York, York, United Kingdom; 2 Department of Entomology, Cornell University, Ithaca, New York, United States of America; French National Centre for Scientific Research – Université Aix-Marseille, France

## Abstract

**Background:**

Animals are chronically infected by benign and beneficial microorganisms that generally promote animal health through their effects on the nutrition, immune function and other physiological systems of the host. Insight into the host-microbial interactions can be obtained by comparing the traits of animals experimentally deprived of their microbiota and untreated animals. *Drosophila melanogaster* is an experimentally tractable system to study host-microbial interactions.

**Methodology/Principal Findings:**

The nutritional significance of the microbiota was investigated in *D. melanogaster* bearing unmanipulated microbiota, demonstrated by 454 sequencing of 16S rRNA amplicons to be dominated by the α-proteobacterium *Acetobacter*, and experimentally deprived of the microbiota by egg dechorionation (conventional and axenic flies, respectively). In axenic flies, larval development rate was depressed with no effect on adult size relative to conventional flies, indicating that the microbiota promotes larval growth rates. Female fecundity did not differ significantly between conventional and axenic flies, but axenic flies had significantly reduced metabolic rate and altered carbohydrate allocation, including elevated glucose levels.

**Conclusions/Significance:**

We have shown that elimination of the resident microbiota extends larval development and perturbs energy homeostasis and carbohydrate allocation patterns of of *D. melanogaster*. Our results indicate that the resident microbiota promotes host nutrition and interacts with the regulation of host metabolism.

## Introduction

It is increasingly recognized that all animals are chronically infected by microorganisms, and that the resident microbiota, especially the substantial microbial community in the alimentary tract, has major effects on nutrient processing, metabolic signaling and, ultimately, the health and well-being of the animal host [1], [2], [3]. There is now persuasive evidence linking the gut microbiota with energy homeostasis of rodent biomedical models and humans, including microbial-mediated promotion of nutrient acquisition and storage [4]. In particular, a causal role of the microbiota in animal energy metabolism is indicated by the elevated lipid levels and other indices of metabolic syndrome in wild-type mice infected with the microbiota from individuals that are obese as a consequence of genetic deficiencies in leptin or Toll-like receptor 5 (a component of the innate immune system that is expressed in the gut) [5], [6].

It is experimentally challenging to study the interactions between the resident microbiota and the nutrition of humans and rodent biomedical models because the microbiota of mammals includes hundreds of taxa, many of which are unculturable, with wide variation in composition among individuals [7], [8], [9]. Simple systems comprising animals bearing one or a few microbial taxa are valuable tools to investigate how resident microorganisms interact with host metabolism [10]. For example, mice experimentally infected with specific bacterial taxa have revealed the effects of the gut microbiota on carbohydrate and energy metabolism [11], [12]. A second approach, adopted in this study, is to use insects that have a less diverse microbiota than mammals, often comprising <20 species [13], [14], [15], [16]. In particular, *Drosophila melanogaster* combines renowned genetic and experimental tractability [17] with a microbiota that is culturable, of low diversity, and uniform among individuals for a given set of conditions. The bacteria associated with *Drosophila* include *Acetobacter*, *Gluconobacter*, *Enterococcus* and *Lactobacillus* [18], [19], [20], [21], [22], [23].

Comparison between animals containing and experimentally deprived of microorganisms is a powerful strategy to investigate the interactions between animals and their resident microbiota. Here, we provide the first analysis of how the resident microbiota affects the organismal physiology of *Drosophila*, with particular emphasis on nutrition. Using insects reared on a diet that supports excellent performance of *Drosophila* with unmanipulated microbiota, we investigate the impact of eliminating the microbiota on host performance (growth, fecundity etc), nutritional status and metabolic rate. Our data suggest that, although the resident microorganisms are not essential for *Drosophila*, they have pervasive effects on the nutrition and metabolic status of their animal host.

**Figure 1:**
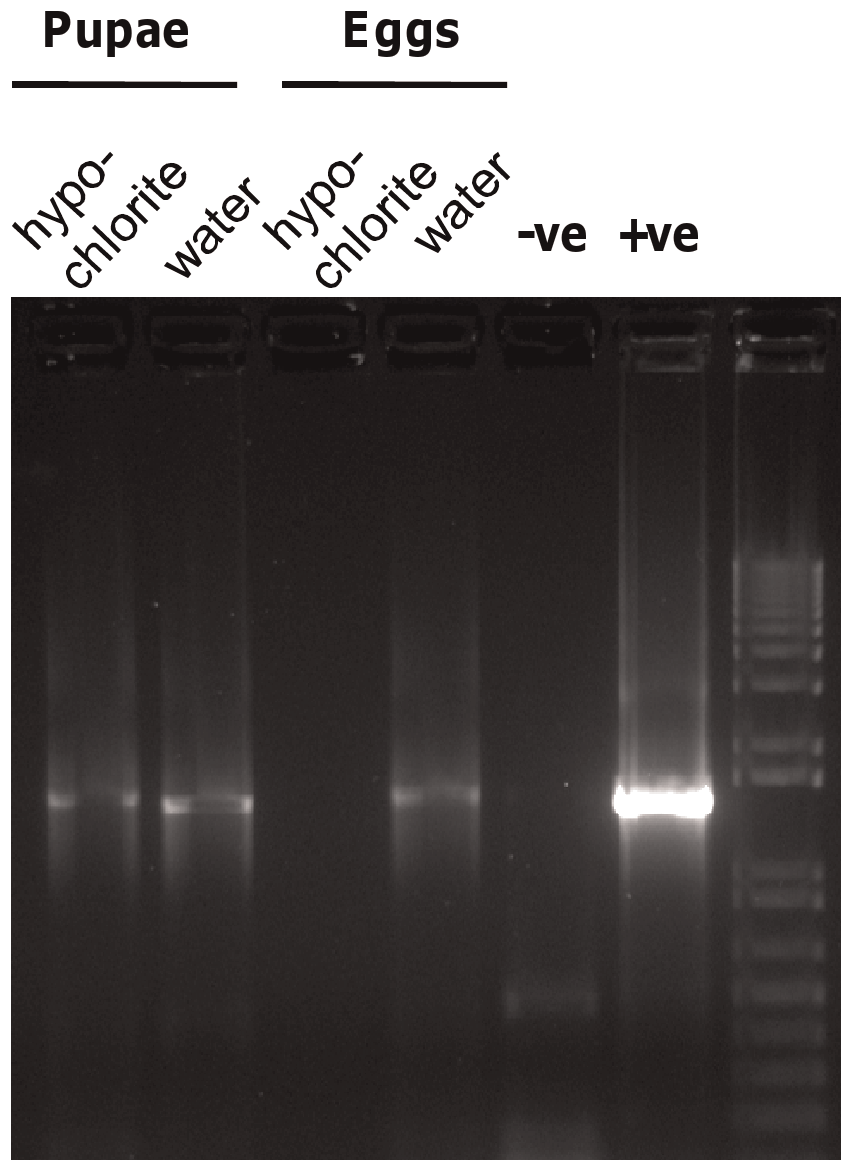
Bacterial complement of *Drosophila*. PCR assay with general 16S rRNA primers of 14-day-old adult flies, derived from pupae washed in 10% sodium hypochlorite solution or sterile water (lanes 1–2), and eggs washed in 10% sodium hypochlorite solution or sterile water (lanes 3–4). Negative and positive controls are PCR reactions with DNA from filtered water and *Drosophila* in standard culture, respectively, as template (lanes 5–6).

## Results

### Bacterial complement of flies

The first experiments tested for the presence of bacteria by PCR with general 16S rRNA gene primers (Figure 1). A PCR product of the predicted size was obtained from flies reared from eggs that had been washed in sterile water (conventional flies) but not from dechorionated eggs (axenic flies). Conventional insects reared to the pupal stage, then surface-sterilized with 10% hypochlorite, and allowed to develop on sterile diet to 14-day-old adults, also bore bacteria.

These data indicate that bacteria are acquired from the external environment by first-instar larvae, and persist through larval development and in internal tissues of pupae to adulthood, validating early studies [24] that quantified CFUs of culturable bacteria without identification. In supplementary PCR assays with general 16S primers throughout the experimental study, axenic flies of all ages invariably yielded negative results, and all conventional flies bore bacteria.

The 454 pyrosequencing of 16S rRNA gene amplicons of DNA from adult flies yielded 46,752 sequence reads with an average length of 352 nucleotides (including the multiplex identifier “MID” and primer sequences), after quality filtering and removal of chimeric sequences. A single cluster with 100% sequence ID to the α-proteobacterium *Acetobacter pomorum* EW816 accounted for 98% of the reads. The remaining reads were assigned to: *Lactobacillus plantarum*, (1.9% of reads) and an uncultured γ-proteobacterium in the family Xanthomonadacae (0.1% of reads) (Table 1).

**Table 1:** 16S rRNA gene amplicons detected by 454 pyrosequencing in 5–7-day-old adult *D.melanogaster*.

NCBI accession number of cluster (this study)	Number of sequence reads		Sequence identity		
	Experimental sample	Reagent-only control	NCBI accession number	Taxonomic identity	% sequence identity
JN592041	45682	26	EU096229.1	*Acetobacter pomorum* strain EW816	100
JN592042	873	7	AL935263.2	*Lactobacillus plantarum* strain WCFS1	100
JN592043	28	1	FJ893035	Uncultured bacterium clone nbt16f09	95.2

### Insect performance

**Figure 2:**
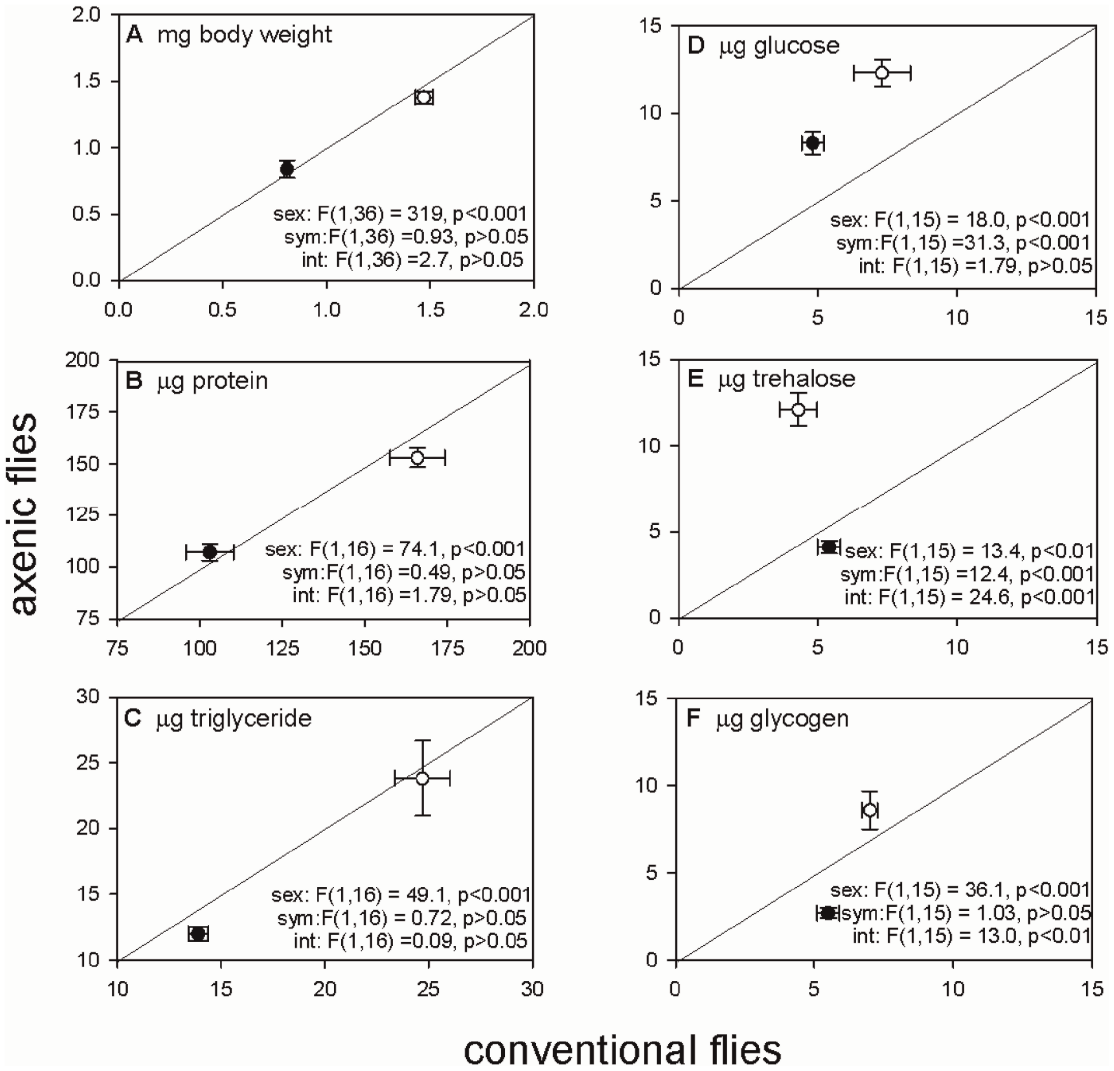
Nutritional indices of 7-to-10-day-old conventional and axenic flies, all expressed on per fly basis. Factors in ANOVA are sex (female ○, male •), sym (conventional or axenic) and int (interaction). Critical probability = 0.008 after Bonferroni correction for six tests. Data are represented as mean +/− SEM.

Table 2 displays the performance indices of conventional and axenic insects. Development time to adulthood was significantly extended by a median value of one day in axenic insects. The other fitness indices tested, survival to adulthood, adult weight, and female fecundity over 7 days, did not differ significantly between the two treatments (Table 2 and Figure 2A).

The basis for the extended development time to adulthood of axenic insects was investigated. The egg dechorionation treatment used to generate axenic insects was confirmed to have no effect on survivorship or development time of the embryos: the median proportion of larvae hatching from dechorionated and control eggs was 0.9 and 0.8, respectively (p>0.05), and median development time to hatching was 19 h for both control and dechorionated eggs (n = 10). The development time of conventional and axenic insects from egg deposition to pupation was 7 and 8 days, respectively (Mann Whitney U, W = 874, p<0.001), the same difference of one day as between development time of conventional and axenic insects from egg deposition to adulthood (Table 2). These data indicate that larval development time was extended in axenic insects.

Two sets of supplementary experiments were conducted. In the first experiment, 10 replicate groups of 10 untreated eggs were transferred to diet containing the antibiotic chlortetracycline at 50 µg ml^−1^, a treatment which reduces the number of culturable bacteria per fly by >90% (Ridley, unpub. data). The median development time to adulthood of the antibiotic-treated flies was 13.5 days (range 12–15 days, n = 10), significantly longer than for conventional flies with median development time of 12 days (range 11–13 days) (Mann Whitney W = 276.5, p<0.001) but not significantly different from axenic flies with median of 13 days and range 12–16 days (Mann Whitney W = 364.5, p>0.05). In the second set of experiments, 10 replicate groups of 10 dechorionated eggs were transferred to sterile diet and sterile diet seeded with feces collected from adult male flies, with untreated eggs as controls. The median development times of the insects reared on the fecal-seeded plates and the conventional flies were identical, at 13 days (range 12–14 days), and significantly shorter than the median development time of axenic flies (14 days, range 13–15 days) (Mann Whitney W = 55, p<0.002). This final analysis was conducted at a different time with a different batch of dietary yeast from the previous experiments, giving slightly different absolute values for development times but the same patterns as shown in Table 2. Taken together, these experiments indicate that the slow development of larvae from dechorionated eggs is caused by the absence of resident microorganisms and could not be attributed to non-specific deleterious effects of the dechorionation procedure.

### Nutritional indices

The values of all nutritional indices (Figure 2) were significantly greater in females than males, reflecting the difference in body size between the sexes. Conventional and axenic flies did not differ significantly in protein or triglyceride contents (Figure 2 B–C), but did vary with respect to the three carbohydrates tested, glucose, trehalose and glycogen. The glucose content was elevated by ca. 70% in both female and male axenic flies (Figure 2D), but the effect of axenic rearing on the trehalose and glycogen content differed significantly between the sexes (Figure 2E–F). For females, the trehalose and glycogen contents of axenic flies were elevated by 68% and 20%, respectively, relative to conventional flies; but these indices were reduced in axenic males, by 30% and 100%, respectively. The sum of glucose, trehalose and glycogen contents was significantly greater in axenic than conventional flies for females (32.1±1.75 µg versus 18.5±1.84 µg per fly, t_6_ = 8.06, p<0.001), but not males (15.9±1.13 versus 15.7±0.71 µg per fly, t_7_ = 0.11, p>0.05).

### Respiration rates

**Figure 3:**
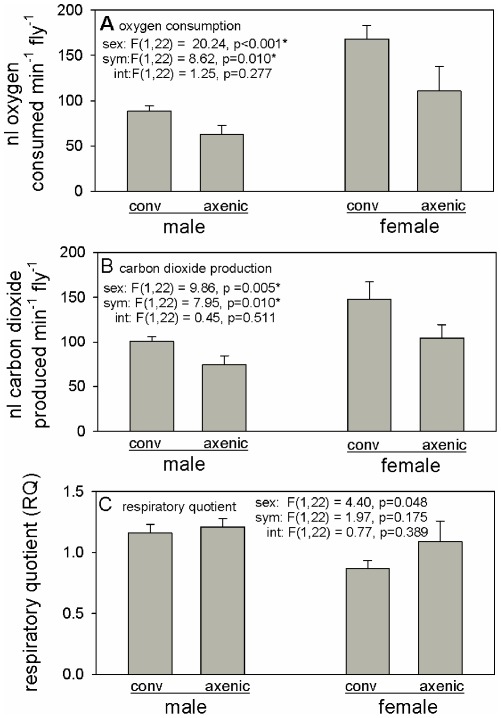
Respiratory exchange of 7-to-10-day-old conventional (conv) and axenic flies. Factors in ANOVA are sex, sym (conventional or axenic) and int (interaction). Critical probability = 0.017 after Bonferroni correction for three tests (*, statistically significant). Data are represented as mean +/− SEM.

For both oxygen consumption and carbon dioxide production, exchange rates were significantly lower in males relative to females, and in axenic flies relative to conventional flies, with non-significant interaction terms in the ANOVA tests (Figure 3A). Bacterial respiration was calculated to contribute <2% to the difference in respiration rate between conventional and axenic flies (Text S1). The RQ was not significantly affected by either sex or treatment (Figure 3B), and the mean values of both males and females did not differ significantly from unity (male RQ: 1.18±0.051 (n = 15), t_14_ = 0.229, p>0.05; female RQ: 0.97±0.087 (n = 11), t_10_ = 0.030, p>0.05), indicating that the dominant respiratory fuel in all flies was glucose.

## Discussion

### Effects of axenic cultivation on *Drosophila* performance

The experimental value of animals deprived of their resident microbiota to study symbiosis function depends critically on the specificity and efficacy of the methods to eliminate the microbiota, and the degree of host dependence on the microbiota. This study demonstrates that axenic *Drosophila* obtained from dechorionated eggs are ideally suited to this approach because egg dechorionation completely eliminates the microbiota (Figure 1), while the eggs are undamaged by the treatment, as indicated by the uniform survivorship and development time of treated and control eggs to hatching, the comparable effects of dechorionation and antibiotic treatment on development time to adulthood, and the equivalent development time of conventional insects and insects from dechorionated eggs provided with bacteria via fecal washings.

The sole performance effect of eliminating the microbiota identified under the conditions tested here was extended larval development time of axenic *Drosophila*. If this effect were replicated under natural conditions, it would be beneficial for *Drosophila* because multiple eggs are deposited onto rotting fruit, such that larvae are in scramble competition for a transient resource. Individuals that develop rapidly are at a competitive advantage and more likely to pupate before exhaustion of the resource [25].

Importantly, the extended larval development time of axenic flies was not accompanied by any difference in adult body size between axenic and conventional flies (Figure 2) under the rearing conditions employed. Thus, axenic larvae take longer than conventional larvae to reach the critical weight at which they are committed to metamorphosis, but they are able to acquire dietary nutrients and convert them into biomass as efficiently as conventional insects once they have passed the critical weight, i.e. during the interval to cessation of growth (ICG). This suggests that microbial effects are particularly important during larval development prior to ICG.

Nevertheless, these results should be extrapolated beyond the specifics of this study with great caution. Although the literature is fragmentary, there are indications that multiple aspects of diet composition, host genotype and the identity of the resident microbiota may influence *Drosophila* performance, potentially in an interactive fashion. For example, elimination of the microbiota has been reported to reduce the lifespan of *Drosophila* reared on diet containing sucrose [21], but this effect was not replicated for flies reared on a diet containing glucose [18]; and the effect of sugar type on the performance of conventional *Drosophila* can vary with both sugar concentration and host genotype [26]. Performance can also vary with the composition of the microbiota, which is influenced by age and immunocompetence of the *Drosophila* [20], [27]. An indication that diet composition can also affect microbial composition comes from the comparison between the microbiota in the young adult flies studied here and a previously-published analysis of the same *Drosophila* strain reared on a diet with higher yeast content. In both studies, the young adult flies bore *Acetobacter* and *Lactobacillus*, but at ratio of 49∶1 in this study (4.8% yeast diet), and 1∶4 in the study using 8.6% yeast diet [20]. Further research involving systematic variation of these multiple factors is required to elucidate the multiway interactions between diet, bacterial composition, host genotype and insect performance.

### Effects of axenic cultivation on the nutritional phenotype of *Drosophila*

A key finding of this study was the impact of the microbiota on the carbohydrate allocation pattern of the adult *Drosophila* (Figure 2D–F). Furthermore, the elevated female-specific body glycogen content and prolonged larval development, obtained for axenic flies on the diet used in this study [with 5∶1 carbohydrate∶protein ratio (5C∶1P)], has also been reported for conventional flies on diets containing 10C∶1P, relative to diets with more balanced C∶P ratios (5C∶1P and 2.5C∶1P) [28]. These data suggest that the bacteria may reduce insect utilization of ingested carbohydrate. Specifically, the bacteria in the gut lumen may compete with the *Drosophila* for ingested carbohydrate. Additionally or alternatively, they may suppress insect digestion of complex dietary carbohydrates. Candidate bacterial products are acetic acid and lactic acid, which are secreted by *Acetobacter* and *Lactobacillus* species, respectively, and are known to reduce the digestibility of starch and other carbohydrates by mammals [29], [30], [31], [32]. The impact of the microbiota on the nutritional status of *Drosophila* may also arise from system-level effects on host signaling networks that regulated carbohydrate allocation patterns. In particular, *Acetobacter* and *Lactobacillus* (both resident in the flies studied here) have been implicated to promote insulin signaling in different *Drosophila* genotypes reared on diets of different formulations from this study [33], [34]. The sex-specific effect of axenic cultivation on the level of glycogen and also the disaccharide blood sugar trehalose in *Drosophila* (Figure 2) is consistent with the prediction that nutrient allocation to energy reserves is more responsive to diet composition in females, which have a high reproductive investment, than in males [35].

Other data suggest that the microbiota has a profound effect on energy homeostasis of *Drosophila*. In particular, the significantly elevated glucose content of axenic flies can be attributed to one or both of reduced demand and increased supply of glucose. Glucose is likely the dominant respiratory fuel for both conventional and axenic flies (RQ does not differ significantly from unity), but axenic flies have a lower respiratory demand for glucose, as indicated by their lower respiration rate than conventional flies. A greater supply of glucose from ingested food for axenic than conventional flies is also predicted (see above). In particular, a contribution of bacterial-derived acetic acid in depressing the glucose content of *Drosophila* is suggested by the evidence that lowered blood glucose levels accompany the reduced digestibility of complex carbohydrates in human volunteers who include acetic acid in their diet [31]. These effects in axenic flies may be linked to reduced insulin/insulin-like growth factor signaling (IIS), which is known to promote free glucose levels [36], alter mitochondrial function resulting in reduced rates of oxygen consumption and oxidative phosphorylation [37], and depress *Drosophila* developmental rate prior to ICG [38]. The absence of any discernible effect of hyperglycemia on the weight or fecundity of axenic flies (Figure 2 and Table 2) reflects the far greater physiological tolerance of variable sugar levels in insects than in mammals [39], [40].

Both diet composition [28] and axenic cultivation (this study) had no effect on the protein density of the flies. This important result is fully consistent with previous evidence that food consumption and nutrient allocation in *Drosophila* are regulated to maintain a certain target protein content [41]. Studies involving *Drosophila* reared on diets with lower protein content and protein∶carbohydrate ratio than used in this study would be required to investigate the role of the microbiota in protein nutrition.

In conclusion, this study demonstrates that the nutritional phenotype of *Drosophila* is strongly influenced by chronic infection with microorganisms that influence energy homeostasis and carbohydrate allocation patterns. These effects are predicted to both accompany and interact with signaling interactions between the microbiota and the host that are known to underpin normal development and cellular homeostasis, especially of the *Drosophila* gut [42]. Although the detail of the relationship between animals and their resident microbiota is anticipated to vary with host and symbiont taxa and environmental circumstances, the *Drosophila* association demonstrates the generality that a comprehensive explanation of the nutritional phenotype of animals requires understanding of the animal interactions with its microbiota.

**Table 2:** Fitness indices of conventional and axenic *Drosophila*.

Treatment	Performance indices Median (range)			
	Survival to adulthood (per 10 eggs)	Development time to adulthood (days)	Number of eggs deposited day^−1^ female^−1^	Male lifespan (days)
Conventional	7.0 (5–8)	12 (11–13)	17.5 (5–24)	41.5 (20–62)
Axenic	7.5 (5–10)	13 (12–16)	17.0 (11.5–22)	49.0 (34–61)
Mann-Whitney U^a^	W = 107, p>0.05	W = 313, p<0.001	W = 103.5, p>0.05	W = 63, p>0.05

a Critical probability = 0.0125, after Bonferroni correction for 4 tests.

## Materials and Methods

### Fly cultures and experimental design

*Wolbachia*-free *Drosophila melanogaster* strain Canton-S was reared at 25°C with a 12 h∶12 h light–dark cycle on autoclaved medium containing 96 g glucose (Sigma), 48 g inactive dry yeast and 14 g agar (both from Genesse Scientific) l^−1^, equivalent to 5∶1 (g/g) carbohydrate∶protein ratio. Experiments were initiated with eggs deposited overnight by mated females. Two egg treatments were used: dechorionated eggs (yielding axenic insects), obtained by washing in sterile deionized water, immersion in 10% sodium hypochlorite solution for 5 min, and then three rinses in sterile water; and control eggs (yielding conventional insects), for which the hypochlorite was replaced by sterile water. To initiate experiments, 10 eggs were transferred to uncrowded conditions comprising replicate vials (2 cm diam.) containing ca. 8 ml diet. All manipulations were conducted in a laminar flow cabinet with aseptic technique. In some experiments, the microbiota was depleted by rearing insects from control eggs on diet supplemented with 50 µg chloretetracycline (Sigma) ml^−1^. To supplement the diet with *Drosophila* microbiota, adult males were cultured for 24 h on sterile medium, which was then rinsed with sterile PBS, and 50 µl of the fecal washing was added to the test diets; the fecal washings were confirmed to contain viable *Acetobacter*, by plating onto bacteriological agar.

### Bacterial content of flies

DNA extractions were conducted with the DNeasy Blood and Tissue Kit (Qiagen, Valencia, California, USA) following a protocol modified from the manufacturer's instructions to ensure disruption of Gram-positive bacteria. Specifically, samples were hand-homogenised in 20 mM Tris-HCl pH 8.0, 2 mM sodium EDTA, 1.2% Triton® X-100 containing 20 mg lysozyme ml^−1^, and the homogenates were incubated at 37°C for 1.5 h with a 5-min bead-beating in a Disruptor Genie® using 0.1 mm glass beads (Scientific Industries) at 45 min.

Individual conventional and axenic flies were checked for the presence of bacteria by PCR using general 16S rRNA gene primers 16SA1: 5′- AGAGTTTGATCMTGGCTCAG-3′ and 16SB1: 5′ – TACGGYTACCTTGTTACGACTT-3′ [43], yielding *ca.* 1.5 kb product (27F-1522R). The PCR reactions contained 1× *Taq* polymerase buffer, 0.24 mM of each dNTP, 2 mM MgCl_2_, 0.32 µM primers, 1 µl template DNA and 0.025 U Platinum *Taq* in 25 µl. The cycling conditions were 5 min at 94°C, followed by one cycle of 1 min at 55°C, 72°C for 2 min and 25–30 cycles of 1 min at 94°C, 1 min at 55°C and 2 min at 72°C with a final incubation of 8 min at 72°C. All experiments included PCR reactions replacing template DNA with water, as negative control. PCR products were separated by electrophoresis in a 1% agarose gel with molecular weight markers, and visualized under ultraviolet light after staining with Sybr Safe (Invitrogen).

The diversity of bacteria associated with the flies was investigated by 454 pyrosequencing of the V2 region of the 16S rRNA gene. Three replicate PCR reactions were conducted on the experimental sample comprising DNA extracted from five pooled 7-day-old adults (3 male and 2 female) conventional flies, with a reagent-only negative controls. The primers were 27F (ACGCTCGACAAGAGTTTGATCMTGGCTCAG) and 338R (TGCTGCCTCCCGTAGGAGT), with the sample-specific 27F primer bearing a multiplex identifier (MID) sequence [MID2 (ACGCTCGACA) for the experimental sample, MID11 (TGATACGTCT) for the control sample) and all 27F and 338R primers modified with 5′-Adaptor A and 5′-Adaptor B sequences (Roche), respectively. The reactions comprised 0.6 U *Platinum*® *Taq* DNA Polymerase (Invitrogen) in 1× PCR buffer, 2 mM MgCl_2_, 8 pmol each primer, 0.24 mM dNTP, and 1 µl template in 25 µl final volume, at 94°C for 10 min followed by 25 cycles of 94°C for 1 min, 58°C for 1 min, and 72°C for 1 min. Following purification with the Qiagen Qiaquick purification kit and quantification using the Quant-iT™ PicoGreen® Kit, each sample was diluted to 1×10̂7 molecules per microliter (based on 350 bp size of the products). Emulsion PCR with 1.5 copies per bead used only “A” beads for unidirectional sequencing on 454 GS-FLX pyrosequencing instrument with standard Titanium chemistry.

Pyrosequencing ﬂowgrams were converted to sequence reads using 454 Life Science software (www.454.com). The data were then processed using Pyrotagger [44] as previously described [20], with minor modifications. In brief, reads with ambiguous nucleotides (N), <290 nucleotides after the forward primer, mismatches with the 16S rRNA gene primers, and all reads with 0.2% per-base error probability (≥3% of bases with Phred scores <27) were removed The remaining sequences were trimmed to 290 nucleotides, dereplicated and clustered into operational taxonomic units (OTUs) with 97% sequence identity (ID) threshold. The most abundant unique sequence of each OTU cluster was selected as representative, and checked for chimeras by the Mallard algorithm (Ashelford et al., 2006). Non-chimaeric sequences was assigned to bacterial taxa by NCBI StandAlone BLAST (megaBLAST program) using the nucleotide (nt) database (13 August 2011) with default settings, and allocated to the experimental or control sample according to the MID sequence. The sequences of the three clusters are available at NCBI, with accession numbers provided in Table 1.

### Insect performance indices

Vials with dechorionated or control eggs were monitored daily, and the pupation and eclosion dates of every insect surviving to adulthood was scored, from which the number surviving to adulthood and median development time per vial was determined. Three days later, 10 females were selected at random from across each treatment for analysis of fecundity. Each insect was transferred aseptically to an individual sterile 15 ml Falcon tube containing autoclaved diet in the lid. The lid was changed daily for 7 days, the number of eggs per lid per day was scored, and the median number of eggs per day deposited by each female was determined.

### Nutritional analyses

Ten replicate 7-to-10-day-old adult flies were weighed on a Mettler MX5 microbalance (1 µg accuracy). The flies were then homogenized in 80 µl ice-cold buffer comprising 10 mM Tris, 1 mM EDTA pH 8.0 and 0.1% (v/v) Triton-X-100 with hand-held homogenizer, and centrifuged at 7,000 *g* at 4°C for 1 min. The supernatant was used for analysis of protein, triglyceride and carbohydrates using coupled colorimetric assays with an xMark™ microplate spectrophotometer, following manufacturer's instructions (5 replicates per assay). The assay kits were the triglyceride assay kit of Sigma (catalogue number TG-5-RB); the Coomassie Brilliant Blue microassay method of BioRad (catalogue number 500-0201), with bovine serum albumin as standard (40–480 µg protein ml^−1^) for protein; and the glucose assay kit of Sigma (catalogue number GAGO20) for glucose and, following trehalase (3.7 U/ml) and amyloglucosidase (2 U ml^−1^) treatment, for trehalose and glycogen, respectively.

### Respirometry

Respiratory oxygen consumption and carbon dioxide production by 7-to-10 day old adult flies were determined by stop-flow respirometry with air scrubbed of water vapour and carbon dioxide by silica/Ascarite columns. All experiments were conducted at 25°C with low light conditions that minimized insect activity, and at 3–7 hours after onset of the light period, with the flies of each treatment analyzed at different times on multiple days, to avoid any confounding effects of circadian rhythm in *Drosophila* respiration rates. Each replicate of 5 flies was transferred to a respirometry chamber comprising a 5 ml syringe, and allowed to acclimate for 30 minutes prior to analysis, by which time they were quiescent. The air in the syringe was then replaced by 3.2 ml dried carbon dioxide-free air, with airflow at 57 ml min^−1^. The carbon dioxide and oxygen content of the syringe was determined 30 minutes later by injecting 3 ml of the syringe volume into Sable Systems SS3 Gas Analyzer Sub-sampler with an FCA-10A CO_2_ analyzer and FC-10 O_2_ Analyzer (Sable systems, Nevada, USA), respectively. The gas analyzers were calibrated with 50 ppm CO_2_ gas and 20.9% O_2_ gas. Carbon dioxide and oxygen contents were analyzed using the Sable System data acquisition software (Expedata, Sable Systems, Nevada, USA). All experiments included an empty baseline chamber, as a control for drift in the baseline measures.

## Supporting Information

Text S1 Predicted contribution of gut bacteria to the respiration rate of *Drosophila*
(DOC)

## References

[1] Kau AL, Ahern PP, Griffin NW, Goodman AL, Gordon JI (2011). Human nutrition, the gut microbiome and the immune system.. Nature.

[2] Diamant M, Blaak EE, de Vos WM (2011). Do nutrient-gut-microbiota interactions play a role in human obesity, insulin resistance and type 2 diabetes?. Obes Rev.

[3] Musso G, Gambino R, Cassader M (2010). Gut microbiota as a regulator of energy homeostasis and ectopic fat deposition: mechanisms and implications for metabolic disorders.. Curr Opin Lipidol.

[4] Venema K (2010). Role of gut microbiota in the control of energy and carbohydrate metabolism.. Curr Opin Clin Nutr Metab Care.

[5] Ley RE, Peterson DA, Gordon JI (2006). Ecological and evolutionary forces shaping microbial diversity in the human intestine.. Cell.

[6] Vijay-Kumar M, Aitken JD, Carvalho FA, Cullender TC, Mwangi S (2010). Metabolic syndrome and altered gut microbiota in mice lacking Toll-like receptor 5.. Science.

[7] Dethlefsen L, McFall-Ngai M, Relman DA (2007). An ecological and evolutionary perspective on human-microbe mutualism and disease.. Nature.

[8] Costello EK, Lauber CL, Hamady M, Fierer N, Gordon JI (2009). Bacterial community variation in human body habitats across space and time.. Science.

[9] Nemergut DR, Costello EK, Hamady M, Lozupone C, Jiang L (2011). Global patterns in the biogeography of bacterial taxa.. Environ Microbiol.

[10] Faith JJ, Rey FE, O'Donnell D, Karlsson M, McNulty NP (2010). Creating and characterizing communities of human gut microbes in gnotobiotic mice.. ISME J.

[11] Mahowald MA, Rey FE, Seedorf H, Turnbaugh PJ, Fulton RS (2009). Characterizing a model human gut microbiota composed of members of its two dominant bacterial phyla.. Proc Natl Acad Sci U S A.

[12] Marco ML, Peters TH, Bongers RS, Molenaar D, van Hemert S (2009). Lifestyle of *Lactobacillus plantarum* in the mouse caecum.. Environ Microbiol.

[13] Dillon RJ, Dillon VM (2004). The gut bacteria of insects: nonpathogenic interactions.. Annu Rev Entomol.

[14] McFall-Ngai M (2007). Adaptive immunity: care for the community.. Nature.

[15] Morales-Jimenez J, Zuniga G, Villa-Tanaca L, Hernandez-Rodriguez C (2009). Bacterial community and nitrogen fixation in the red turpentine beetle, *Dendroctonus valens* LeConte (Coleoptera: Curculionidae: Scolytinae).. Microb Ecol.

[16] Robinson CJ, Schloss P, Ramos Y, Raffa K, Handelsman J (2010). Robustness of the bacterial community in the cabbage white butterfly larval midgut.. Microb Ecol.

[17] Ashburner M (1989). *Drosophila*, A Laboratory Handbook Cold Spring Harbor: Cold Spring Harbor Press.

[18] Ren C, Webster P, Finkel SE, Tower J (2007). Increased internal and external bacterial load during *Drosophila* aging without life-span trade-off.. Cell Metab.

[19] Roh SW, Nam YD, Chang HW, Kim KH, Kim MS (2008). Phylogenetic characterization of two novel commensal bacteria involved with innate immune homeostasis in *Drosophila melanogaster*.. Appl Environ Microbiol.

[20] Wong C-N, Ng P, Douglas AE (2011). Low diversity bacterial community in the gut of the fruitfly *Drosophila melanogaster*.. Environmental Microbiology.

[21] Brummel T, Ching A, Seroude L, Simon AF, Benzer S (2004). *Drosophila* lifespan enhancement by exogenous bacteria.. Proc Natl Acad Sci U S A.

[22] Corby-Harris V, Pontaroli AC, Shimkets LJ, Bennetzen JL, Habel KE (2007). Geographical distribution and diversity of bacteria associated with natural populations of *Drosophila melanogaster*.. Appl Environ Microbiol.

[23] Cox CR, Gilmore MS (2007). Native microbial colonization of *Drosophila melanog*aster and its use as a model of *Enterococcus faecalis* pathogenesis.. Infect Immun.

[24] Bakula M (1969). The persistence of a microbial flora during postembryogenesis of *Drosophila melanogaster*.. J Invertebr Pathol.

[25] Nunney L (1990). *Drosophila* on oranges: colonization, competition, and coexistence.. Ecology.

[26] Reed LK, Williams S, Springston M, Brown J, Freeman K (2010). Genotype-by-diet interactions drive metabolic phenotype variation in *Drosophila melanogaster*.. Genetics.

[27] Ryu JH, Ha E-M, Oh C-T, Seol J-H, Brey PT (2006). An essential complementary role of NF-κB pathway to microbicidal oxidants in *Drosophila* gut immunity.. EMBO Journal.

[28] Matzkin LM, Johnson S, Paight C, Bozinovic G, Markow TA (2011). Dietary protein and sugar differentially affect development and metabolic pools in ecologically diverse *Drosophila*.. J Nutr.

[29] Brighenti F, Castellani G, Benini L, Casiraghi MC, Leopardi E (1995). Effect of neutralized and native vinegar on blood glucose and acetate responses to a mixed meal in healthy subjects.. Eur J Clin Nutr.

[30] Ogawa N, Satsu H, Watanabe H, Fukaya M, Tsukamoto Y (2000). Acetic acid suppresses the increase in disaccharidase activity that occurs during culture of caco-2 cells.. J Nutr.

[31] Johnston CS, Steplewska I, Long CA, Harris LN, Ryals RH (2010). Examination of the antiglycemic properties of vinegar in healthy adults.. Ann Nutr Metab.

[32] Ostman EM, Nilsson M, Elmstahl HGM, Molin G, Bjorck IME (2002). On the effect of lactic acid on blood glucose and insulin responses to cereal products: mechanistic studies in healthy subjects and in vitro.. Journal of Cereal Science.

[33] Shin SC, Kim S-H, You H, Kim B, Kim AC (2011). *Drosophila* microbiome modulates host developmental and metabolic homeostasis via insulin signaling.. Science.

[34] Storelli G, Defaye A, Erkosar B, Hols P, Royet J (2011). *Lactobacillus plantarum* promotes *Drosophila* systemic growth by modulating hormonal signals through TOR-dependent nutrient sensing.. Cell Metabolism.

[35] Boggs CL (2009). Understanding insect life histories and senescence through a resource allocation lens.. Functional Ecology.

[36] Teleman AA (2010). Molecular mechanisms of metabolic regulation by insulin in *Drosophila*.. Biochemical Journal.

[37] Stump CS, Short KR, Bigelow ML, Schimke JM, Nair KS (2003). Effect of insulin on human skeletal muscle mitochondrial ATP production, protein synthesis, and mRNA transcripts.. PNAS.

[38] Edgar BA (2006). How flies get their size: genetics meets physiology.. Nat Rev Genet.

[39] Chapman RF (1999). The Insects: Structure and Function..

[40] Zhang H, Liu J, Li CR, Momen B, Kohanski RA (2009). Deletion of *Drosophila* insulin-like peptides causes growth defects and metabolic abnormalities.. Proc Natl Acad Sci U S A.

[41] Lee KP, Simpson SJ, Clissold FJ, Brooks R, Ballard JW (2008). Lifespan and reproduction in *Drosophila*: New insights from nutritional geometry.. Proc Natl Acad Sci U S A.

[42] Buchon N, Broderick NA, Chakrabarti S, Lemaitre B (2009). Invasive and indigenous microbiota impact intestinal stem cell activity through multiple pathways in *Drosophila*.. Genes Dev.

[43] Fukatsu T, Nikoh N (1998). Two intracellular symbiotic bacteria from the mulberry psyllid *Anomoneura mori* (Insecta, Homoptera).. Appl Environ Microbiol.

[44] Kunin V, Hugenholtz P (2010). PyroTagger: a fast, accurate pipeline for analysis of rRNA amplicon pyrosequence data.. Open J.

