# Supplementary material for: Impact of the Resident Microbiota on the Nutritional Phenotype of Drosophila melanogaster
Source: PLoS One. 2012 May 7;7(5):e36765. doi: 10.1371/journal.pone.0036765 (PMC3346728; doi:10.1371/journal.pone.0036765)
Supplement: Text S1 — Predicted contribution of gut bacteria to the respiration rate of Drosophila (DOC) [file pone.0036765.s001.doc]

**Text S1** Predicted contribution of gut bacteria to the respiration rate of *Drosophila*

A simple calculation establishes that bacterial respiration cannot account for the difference between the respiration rates of conventional and axenic flies.

Let us assume that the gut bacteria in *Drosophila* respire aerobically at the same rate as published values for *E. coli* cells growing exponentially on glucose-medium at 37^o^C.

Published value of *E. coli* respiration rate = 20 mmols oxygen per h per g dry weight [1].

This is equivalent to 4.2 x 10^-6^ nl oxygen per minute per cell

[total dry weight per *E. coli* cell is 2.8 x 10^-13^ g [2]; density of oxygen = 1.43 g l^-1^]

On average, each fly contains 10^4^-10^5^ bacterial cells [3], contributing 0.4 nl oxygen min^-1^ fly^-1^

The difference between the mean rate of oxygen consumption of conventional and axenic flies was 26 and 57 nl min^-1^ fly^-1^ for males and females, respectively (Figure 3).

**On average, the complement of bacteria contributes 1.5% of the total oxygen consumed by the flies.**

1. Andersen KB, von Meyenburg K (1980) Are growth rates of Escherichia coli in batch cultures limited by respiration? J Bacteriol 144: 114-123.

2. Niedhardt FC, ingraham JL, Schaechter M (1990) Physiology of the Bacterial Cell. Sunderland, Massachusetts: Sinauer Associates.

3. Ridley EV (2011) The Impact of Chlortetracycline on Drosophila melanogaster and Aedes aegypti. York: University of York.
